# Phenolic Profile and Antioxidant Activity of Chocolates Supplemented with Bioactive Ingredients

**DOI:** 10.3390/foods15111831

**Published:** 2026-05-22

**Authors:** Paulo Henrique da Silva Santos, Cristina Kaori Suzuki, Suzana Caetano da Silva Lannes, Artur Figueirinha, Fernando Ramos

**Affiliations:** 1 School of Pharmaceutical Sciences, University of São Paulo, São Paulo 05508-000, Brazil; paulo_santos@usp.br (P.H.d.S.S.); cristinasuzuki@usp.br (C.K.S.); 2 Faculty of Pharmacy, University of Coimbra, REQUIMTE/LAQV, Pólo das Ciências da Saúde, Azinhaga de Santa Comba, 3000-548 Coimbra, Portugal; amfigueirinha@ff.uc.pt

**Keywords:** chocolate, phenolic compounds, antioxidant activity, bioactive ingredients

## Abstract

The growing demand for functional foods has stimulated the development of chocolate matrices enriched with bioactive ingredients. This study aimed to evaluate the effect of extraction conditions and formulation strategies on the phenolic profile and antioxidant activity of dark chocolate. Five formulations were evaluated: control chocolate (C), chocolate containing vitamin microcapsules (T1), chocolate with DHA/EPA microcapsules (T2), lipid-modified chocolate with structuring oil (T3), and chocolate combining microcapsules with lipid modification (T4). Phenolic compounds were extracted using hydro-organic solvents of different polarities (50% ethanol, 70% methanol, and 70% acetone). Among the tested solvents, 70% methanol showed the highest extraction efficiency, enabling broader detection of phenolic compounds and alkaloids. HPLC-DAD analysis revealed compounds characteristic of cocoa matrices, including epicatechin, gallic acid, vanillin, and procyanidins, as well as the methylxanthines theobromine and caffeine. Among the formulations, T4 exhibited a greater abundance of extractable compounds and the most complex chromatographic profile. Antioxidant activity was evaluated using DPPH radical scavenging and β-carotene/linoleic acid bleaching assays. T4 also showed the highest antioxidant performance in both assays. These findings suggest that the combination of microencapsulation and lipid phase modification may enhance the extractability and functional expression of bioactive compounds, supporting the development of functional chocolate products with added value.

## 1. Introduction

The rising demand for functional foods has stimulated the development of food matrices enriched with added bioactive compounds capable of providing health benefits. In this context, chocolate stands out not only for its wide sensory acceptance and indulgent nature, but also for its intrinsic composition rich in naturally occurring phenolic compounds such as epicatechin, procyanidins, and gallic acid, which are recognized for their antioxidant, anti-inflammatory, and cardioprotective properties and their potential role in reducing oxidative stress and supporting metabolic health [1].

Due to its unique properties, chocolate can simultaneously act as a natural source of polyphenols and as a delivery system for microencapsulated lipophilic micronutrients, such as vitamins E and D, zinc, and the omega-3 fatty acids eicosapentaenoic acid (EPA) and docosahexaenoic acid (DHA), thereby expanding its potential as a functional food [1,2,3].

In Brazil, chocolate consumption is high and culturally widespread. Data from the *Pesquisa de Orçamentos Familiares* (POF 2017–2018), conducted by the Brazilian Institute of Geography and Statistics -IBGE, revealed that over 75% of Brazilian households consume chocolate products, mainly in industrialized forms such as bars, bonbons, and chocolate powders [4]. This widespread consumption highlights both the cultural and economic relevance of chocolate and its potential as a carrier for bioactive compounds, particularly in nutritional reformulation strategies [5].

However, the extractability and stability of chocolate polyphenols are strongly influenced by the food matrix. The lipid phase, predominantly composed of cocoa butter, plays a central role in the solubilization, protection, and release of these compounds during processing and throughout product shelf life [6,7]. For this reason, structural modifications of the lipid fraction, such as partial replacement with oils rich in bioactive fatty acids or structuring oleogel systems, have been investigated as strategies to preserve and enhance the functionality of phenolic compounds in chocolate by modifying the physicochemical environment and mass transfer properties of the system [5,8,9].

Additionally, technologies such as microencapsulation of micronutrients, including fat-soluble vitamins and polyunsaturated fatty acids, have been widely studied as strategies to improve the oxidative and thermal stability of these ingredients and to modulate their release in food matrices, contributing to the preservation of their functional activity in the final product. In this context, the present study evaluates the impact of incorporating microencapsulated lipophilic micronutrients into a chocolate matrix on the extractable phenolic profile and in vitro antioxidant activity [10,11,12].

In this context, the characterization of phenolic compounds and the evaluation of antioxidant activity in chocolates are essential to support functional claims and potential preventive applications. Chromatographic methods, particularly high-performance liquid chromatography coupled with diode array detection (HPLC-DAD), provide high sensitivity and specificity in identifying these compounds, while complementary in vitro assays, such as DPPH radical scavenging and the *β*-carotene/linoleic acid system, yield relevant parameters regarding the biological functionality of the analyzed systems [5,13,14,15].

To address these aspects in a systematic manner, different chocolate formulations were designed to isolate and evaluate the effects of specific technological strategies. The experimental design included: a control formulation (C), chocolate containing microencapsulated vitamins (T1), chocolate containing microencapsulated omega-3 fatty acids (DHA/EPA) (T2), chocolate with lipid phase modification through oleogel structuring (T3), and a combined formulation incorporating both microencapsulation and lipid modification (T4). This approach enables the assessment of the individual contribution of each strategy as well as their potential synergistic effects on the extractability and functional performance of bioactive compounds within the chocolate matrix.

Therefore, this study aimed to compare and optimize the extraction of phenolic compounds and alkaloids from chocolates using hydro-organic solvents of different polarities, in order to maximize the recovery and detection of these compounds by HPLC-DAD; to qualitatively characterize the phenolic and alkaloid profiles of the different formulations; and to evaluate whether fortification with microencapsulated vitamins E and D, zinc, and the omega-3 fatty acids EPA and DHA, alone or in combination with lipid phase modification, increases the extractable bioactive compound content and in vitro antioxidant activity.

## 2. Materials and Methods

### 2.1. Preparation of Chocolate Formulations

For this study, five dark chocolate formulations were developed, each with a total cocoa content of approximately 60% (comprising cocoa liquor and cocoa butter), with modifications in the lipid phase composition and the inclusion of microencapsulated lipophilic micronutrients. Table 1 presents the percentage composition of the ingredients used in each sample. The raw materials included cocoa liquor and cocoa butter (IBC-Indústria Brasileira de Cacau, Rio das Pedras, Brazil), refined sugar (União, São Paulo, Brazil), soy lecithin and polyglycerol polyricinoleate (PGPR) (Tovani, São Paulo, Brazil), powdered vanillin flavoring (Mix, Jaraguá do Sul, Brazil), and hydroxypropylmethylcellulose (HPMC) (Metachem, São Paulo, Brazil). The samples were designated as follows: C (Control), standard chocolate without microcapsules or lipid replacement; T1, chocolate with microcapsules containing vitamins E, D, and zinc; T2, chocolate with microcapsules containing polyunsaturated fatty acids (DHA and EPA) and high-oleic peanut oil incorporated into the lipid phase; T3, chocolate with 30% of cocoa butter replaced by a structuring oleogel composed of 47% (*w*/*w*) Brazil nut oil, 1.5% (*w*/*w*) HPMC, and distilled water (as detailed in Section 2.3); and T4, chocolate with structuring oleogel combined with both types of microcapsules (vitamins E, D, and zinc; DHA and EPA with high-oleic peanut oil).

The amounts of cocoa butter used in the samples were calculated to ensure that the final product would have a lipid content of 35%, thereby composing a standard dark chocolate with 60% cocoa solids.

### 2.2. Chocolate Production

The production of the samples was carried out at the Food Technology Laboratory III of the Faculty of Pharmaceutical Sciences, University of São Paulo. Manufacturing was performed using a universal mixer with a ball mill, model WA-FA20 (Mazzetti, Tribiano, Italy), which integrates the processes of mixing, refining, and conching into a single system, thereby optimizing space, time, and energy. The ingredients were sequentially added to the equipment at 45 °C. After processing, the chocolates were manually tempered on a marble slab, molded into appropriate forms, and cooled at 5 ± 3 °C for 20 min. Subsequently, they were demolded and stored for further analysis.

### 2.3. Oleogel Preparation

The oleogel was prepared according to the method described by Espert et al. [5,8], with adaptations by Santos, Suzuki and Lannes [5]. The emulsion consisted of 47% (*w*/*w*) Brazil nut oil, 1.5% (*w*/*w*) HPMC, and distilled water to complete 100% (*w*/*w*). Initially, HPMC was dispersed in the oil under mechanical stirring (Fisaton, São Paulo, Brazil) at 280 rpm for 5 min. Subsequently, chilled water (10 °C) was added to promote cellulose hydration, and the mixture was homogenized using an Ultra-Turrax (Marconi, Piracicaba, Brazil), disperser S18N-19G, first at 6500 rpm for 1 min and then at 17,500 rpm for 3 min. The resulting white, viscous emulsion was poured into aluminum trays (40 × 20 cm) and dried in an adiabatic oven at 60 °C for approximately 48 h until a moisture content below 5% was reached.

### 2.4. Extraction of Polyphenols and Alkaloids

The extraction of phenolic compounds and alkaloids was carried out based on the method of Sánchez-Rabaneda et al. [6] with modifications. Chocolate samples (5 g), previously ground (300 µm), were defatted with 40 mL of hexane under agitation for 2 min (Ultra-Turrax, Ystral, Ballrechten-Dottingen, Germany). After centrifugation at 500 rpm for 5 min at 15 °C (3-16K centrifuge, Sigma, Osterode, Germany), the solid residue was used for extraction.

The extraction was performed using three hydro-organic solvents of different polarities to optimize the recovery of phenolic compounds with varying polarities: 70% methanol (70Me) (polarity index: 0.762), 50% ethanol (50Et) (polarity index: 0.654), and 70% acetone (70Ac) (polarity index: 0.355), representing a range from moderate to low polarity in water–organic systems. For each solvent, 30 mL were added to the pellet, followed by vortex mixing (ZX3, Velp Scientifica, Usmate, Italy) for 3 min and sonication in an ultrasonic bath (Sonorex RK510S, Bandelin, Berlin, Germany) for 10 min at 25 °C. The samples were centrifuged again under the same conditions, and the supernatants were collected.

This procedure was repeated twice with the same residue. The obtained extracts were combined and concentrated under reduced pressure in a rotary evaporator (R-300, Büchi, Flawil, Switzerland). The dried residue (approximately 30 ± 5 mg) was reconstituted in 10 mL of methanol, filtered through a 0.45 µm membrane, and diluted 1:10 in methanol–water (50:50, *v*/*v*) prior to HPLC analysis.

### 2.5. HPLC-DAD Analysis of Polyphenols and Alkaloids

Polyphenol and alkaloid profiling was carried out using a high-performance liquid chromatography system coupled to a diode array detector (HPLC-DAD model VC-D11-A, Vanquish; Thermo Fisher Scientific, Waltham, MA, USA), equipped with a C18 UHPLC column (Vanquish, Thermo Fisher Scientific, Waltham, MA, USA), maintained at 25 °C. The mobile phase consisted of water containing 0.1% formic acid (Phase A) and methanol containing 0.1% formic acid (Phase B). The acidic mobile phase system was selected to enhance chromatographic separation and peak definition of phenolic compounds across a wide polarity range.

The elution gradient started at 95% Phase A and 5% Phase B, reaching 70% A and 30% B within 5 min. At 24 min, the system achieved 100% Phase B and was returned to the initial conditions at 30 min to ensure column re-equilibration. The flow rate was set at 1.0 mL/min, and the injection volume was 10 µL. This gradient program allowed the sequential elution of more polar phenolic acids followed by semi-polar flavonoids and more lipophilic compounds, providing a representative chromatographic fingerprint of cocoa-derived phenolics.

Compound identification was performed based on retention time (RT) and UV–Vis spectral characteristics obtained from the diode array detector, compared with commercial analytical standards (epicatechin, gallic acid, and procyanidin B2; Sigma-Aldrich, St. Louis, MO, USA) analyzed under identical chromatographic conditions. Detection wavelengths were selected according to the characteristic absorption maxima of the target compounds, namely 280 nm for catechin, epicatechin, and procyanidins, and 270 nm for gallic acid.

The HPLC-DAD analysis was conducted for qualitative and comparative purposes, aiming to characterize the extractable phenolic profile and evaluate differences among chocolate formulations. Relative comparisons between samples were based on chromatographic profiles and peak area distribution, allowing the assessment of formulation-dependent effects on phenolic extractability. Data acquisition and processing were performed using Chromeleon™ Data System software(version 7.3.2, Thermo Fisher Scientific, Waltham, MA, USA), with peak integration carried out consistently across all samples to ensure comparability of the chromatographic fingerprints.

### 2.6. Determination of Antioxidant Capacity

#### 2.6.1. DPPH Radical Scavenging Assay

Antioxidant capacity was assessed using the 2,2-diphenyl-1-picrylhydrazyl (DPPH•) radical scavenging method, adapted from Moure et al. [16]. Samples were diluted to 0.5 mg/mL; 50 μL of each sample were mixed with 2 mL of a methanolic DPPH• solution (14.2 μg/mL). The reaction mixture was maintained in the dark for 30 min, and absorbance was measured at 517 nm using a UV-vis spectrophotometer (U-3900, Hitachi, Tokyo, Japan). Results were expressed as inhibition percentage (IP%), Trolox equivalents (μg TE/mL), and EC50 (mg/mL), defined as the concentration required to inhibit 50% of DPPH radical.

#### 2.6.2. β-Carotene Bleaching Assay

The assay followed Miller [17] with modifications. An antioxidant emulsion was prepared with 2 mg *β*-carotene dissolved in chloroform, 40 mg linoleic acid, 400 mg Tween^®^ 40, and 100 mL oxygenated water, homogenized and evaporated at 40 °C. Samples (0.2 mL at 5 mg/mL) were added to 5 mL of emulsion and incubated at 50 °C for 2 h. Absorbance was recorded at 380 nm at 0 and 120 min. Antioxidant activity was expressed as the Antioxidant Activity Coefficient (AAC), calculated from the absorbance difference between sample and control.

## 3. Results

Extraction using 70% methanol showed the widest detection range: 18 of the 19 monitored compounds appeared in at least four of the five formulations (Table 2). The chromatographic profile revealed a dense array of peaks between 3 and 18 min, in addition to several late-eluting peaks (>22 min). This result confirms the long-standing use of hydro-methanolic solvents as the gold standard for extracting polar and semi-polar phytochemicals [18,19].

At extraction in 50% ethanol (Table 3), a reduction in the number of detected compounds was observed compared with methanolic extraction. Several peaks present in methanol extracts were not detected under ethanolic conditions, indicating differences in extraction efficiency related to solvent polarity and matrix interactions [20].

Ac70 (Table 4) showed poor extraction performance, with few compounds detected and poorly resolved peaks. Its low polarity and aprotic character limit the extraction of phenolics in fat-rich matrices such as chocolate. Although useful for dried plant materials, its effectiveness is reduced in lipophilic and structured systems, such as formulations containing oleogel or microcapsules, and therefore its use should be avoided or carefully adjusted [18,19,20].

**Figure 1:**
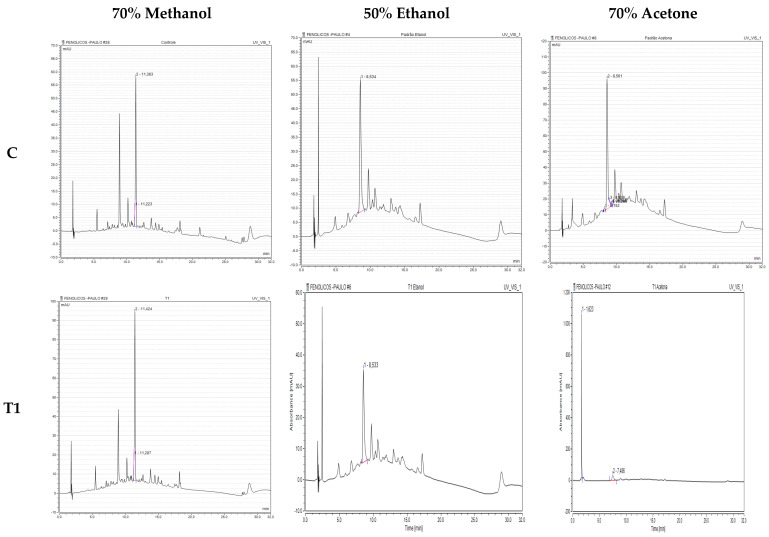

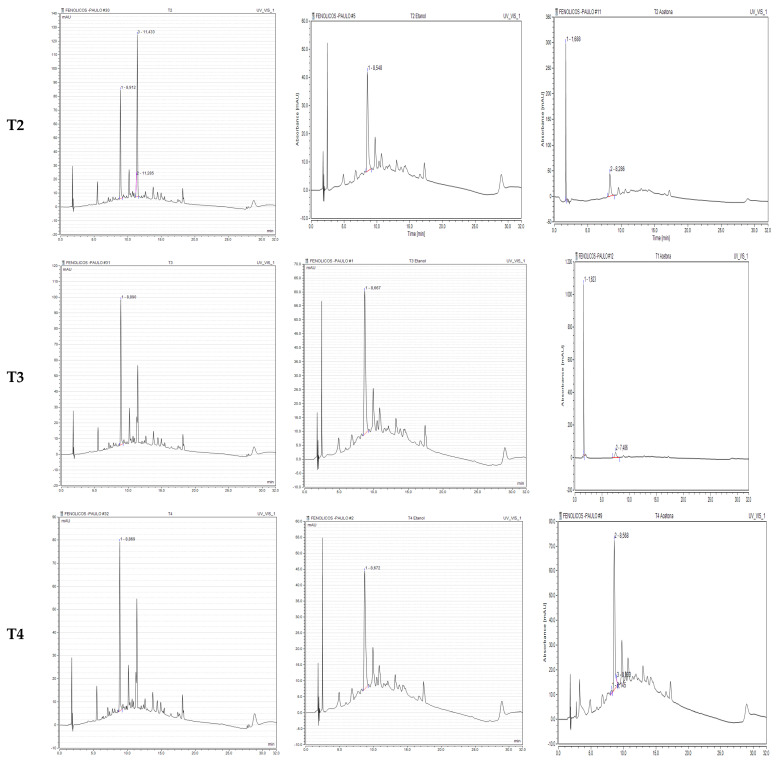
HPLC-DAD chromatograms (detection at 280 nm) of chocolate extracts obtained with different solvents (70% methanol, 50% ethanol, and 70% acetone). Samples: C (control), T1 (vitamin D, E and zinc microcapsules), T2 (DHA/EPA), T3 (oleogel), T4 (microcapsules + oleogel).

Figure 1 visually summarizes the impact of solvent selection and matrix reformulation on the chromatographic profiles. Methanolic extracts exhibited the highest peak density and signal intensity across the retention range, particularly between 4 and 18 min. In contrast, ethanol and acetone extracts showed reduced complexity and lower signal intensity, indicating limited extraction of certain phenolic compounds.

These differences are associated with both solvent physicochemical properties and structural modifications in the chocolate matrix, which may influence compound release and solubilization [20].

The class-based analysis of compounds (Table 5) provides a clearer overview of extraction patterns and possible underlying mechanisms. Phenolic acids (RT 4–9 min), such as gallic, caffeic, and ferulic acids, showed higher extractability in methanolic systems, intermediate performance with ethanol, and were almost completely absent in acetone extracts.

This trend may be associated with the high density of hydroxyl groups in these compounds, which increases their affinity for polar, protic solvents capable of stabilizing ionized species. In contrast, lower recoveries observed in acetone may be related to its lower polarity and aprotic nature [18,19,20].

### Antioxidant Activity

Table 6 presents the antioxidant activity determined by DPPH radical scavenging and β-carotene bleaching assays. Significant differences were observed among formulations (*p* < 0.05), with T3 and T4 showing higher activity compared to the control and other treatments.

## 4. Discussion

### 4.1. Phenolic Profile

The qualitative evaluation of methanolic, ethanolic, and acetonic extracts obtained from the five chocolate formulations (C, T1–T4) revealed marked differences in the ability of each solvent to solubilize phenolic compounds and alkaloids. The detailed presence/absence data shown in Table 2, Table 3 and Table 4—complemented by the summary in Table 5 and the chromatograms in Figure 1—provide a comprehensive overview of the selectivity of each solvent, as well as the possible effects of structural and compositional changes in the chocolate matrix on phenolic solubilization, and the consistency of the findings with contemporary literature.

First, the Me70 extract stood out by enabling the detection of virtually all monitored analytes. This performance may be associated with the high polarity of hydro-methanolic systems, which favor the extraction of phenolic compounds commonly present in cocoa matrices [18,19]. The chromatographic response (Figure 1) displayed a dense array of peaks between 3 and 18 min, indicating the presence of several compounds with different polarities extracted under these conditions. These findings support the suitability of hydro-methanolic solvents for recovering a wide range of phenolic compounds from cocoa-based products [18,19].

In contrast, the Et50 extract showed a marked reduction in the number and intensity of peaks, suggesting lower extraction efficiency compared with the methanolic system [20]. On the other hand, extraction using 70% acetone (Ac70) showed limited performance, with fewer detected compounds and poorly resolved peaks, which may indicate co-extraction of interfering lipids and reduced compatibility with polyhydroxylated phytochemicals present in cocoa matrices [19,20].

Beyond solvent composition, the structural characteristics of the chocolate matrix may also influence the extractability of phytochemicals. Formulations containing oleogel (T3) exhibited differences in the chromatographic profile compared with the control formulation, which may be related to modifications in the lipid network organization introduced by the oleogel system. Such structural changes can influence the interaction between phenolic compounds and the lipid phase, potentially affecting their release during solvent extraction [6].

In formulations containing vitamin microcapsules (T1) or DHA/EPA microcapsules (T2), variations in the chromatographic profiles were also observed, suggesting that encapsulating matrices may influence the accessibility of phenolic compounds. Polysaccharide-based encapsulating systems, such as those used in T1, may interact differently with polar phytochemicals compared with lipid–protein microcapsules such as those present in T2, which can modify the partition behavior of these compounds during extraction [21]. The hybrid formulation (T4), combining oleogel and microencapsulation strategies, presented a more complex chromatographic profile compared with other reformulated samples, reinforcing the importance of considering matrix–compound interactions when evaluating functional chocolates developed with multiple technological approaches.

These results illustrate how the solvent–matrix interaction may influence extraction efficiency in functional chocolates. Simple ingredient substitutions—such as the introduction of oleogels or microcapsules—can alter the spatial distribution and solubility of phenolic compounds within the matrix, directly impacting HPLC profiles. This observation highlights the importance of considering structural modifications when developing extraction protocols for reformulated chocolate systems, particularly when functional or nutritional claims are assessed [6,9]. Recent studies indicate that encapsulating structures or lipid networks can significantly modify diffusional behavior and intermolecular interactions of phenolics within the matrix, suggesting that extraction methodologies may need to be tailored to each case [19,20]. Therefore, qualitative analyses that do not consider these structural aspects may lead to under- or overestimation of certain phytochemicals. Such findings underscore the importance of integrated analytical strategies adapted to the physicochemical characteristics of reformulated systems, particularly in products with functional claims and nutraceutical appeal [18].

Differences among the formulations C (control), T1 (vitamin D, E and zinc microcapsules), T2 (DHA/EPA microcapsules), T3 (oleogel), and T4 (microcapsules + oleogel) were clearly reflected in the HPLC-DAD chromatographic profiles, revealing that matrix structural modifications may affect the accessibility and selective extraction of phenolic compounds across solvent systems.

Formulation T2 (DHA/EPA microcapsules) showed a noticeable reduction in the number and intensity of peaks in the early chromatographic region (approximately 4–9 min), typically associated with phenolic acids, when compared with the control formulation in both methanolic and ethanolic extracts. This pattern may suggest that the presence of lipid-rich microcapsules can influence the accessibility of certain polar phenolic compounds during solvent extraction, an effect that became more evident when less polar solvent systems were used. In contrast, formulation T3 (oleogel) presented chromatographic profiles more similar to the control when polar solvents were applied, particularly within retention time regions associated with phenolic compounds, suggesting that structural modifications in the lipid phase may influence the release of these compounds during extraction. However, this tendency was less evident in less polar solvents. Additionally, signals consistent with aromatic aldehyde derivatives, such as vanillin, were observed in the chromatographic profile, indicating the presence of other phenolic-related compounds commonly found in cocoa matrices [2,5].

Formulation T4 (microcapsules + oleogel combination) exhibited an apparent increase in extract complexity across solvent systems, with chromatographic profiles approaching control levels in methanol and ethanol. This behavior may suggest a combined effect of matrix structuring and encapsulation on compound accessibility, although the underlying mechanisms were not directly evaluated in the present study. Flavan-3-ols, represented by epicatechin (RT 9–14 min), were detected mainly in methanolic extracts, while absent in acetone extractions and partially compromised in ethanol, especially in sample T2. The absence of catechin in T2-Et may be associated with possible interactions with the DHA/EPA encapsulating matrix, which could limit its diffusion into the extractive medium [20,21].

The class of flavonols and flavones (RT 14–18 min), including quercetin, kaempferol, and luteolin, demonstrated an even stronger dependence on solvent type and matrix modification. These compounds possess multihydroxylated aromatic structures and high dipole moments, making them better extracted with methanol. In ethanol, only quercetin was detected in T3, suggesting that the oleogel may contribute to greater release of these molecules, possibly by modifying the lipid matrix structure and facilitating diffusion [15,20]. The complete absence of these classes in acetonic extracts once again highlights the limitations of this solvent in fatty systems.

Finally, compounds eluting in the late chromatographic region (RT > 22 min), together with early signals between 3 and 5 min, complete the overall chromatographic profile observed for the chocolate samples. These observations highlight that the extraction and detection of bioactive compounds are strongly influenced not only by the chemical characteristics of the solutes, but also by the physicochemical structure of the chocolate matrix and the solvent system used during extraction [23].

### 4.2. Antioxidant Activity and Influence of the Lipid Matrix

The antioxidant capacity of the five chocolate formulations—Control, T1 (vitamin E, D, and Zn microcapsules), T2 (DHA/EPA microcapsules), T3 (oleogel), and T4 (oleogel + microcapsules)—was evaluated using the DPPH• radical scavenging assay (µmol TE g^−1^) and the β-carotene bleaching assay (inhibition %) (Table 6). The protocols followed Brand-Williams, Cuvelier, and Berset [24] for DPPH and Deghima et al. [25] for β-carotene, complementary methods that measure electron and hydrogen transfer mechanisms [26,27].

According to studies by Todorovic et al. [23], milk chocolates rarely exceed 40 µmol TE g^−1^, a value consistent with the control in this study. The addition of isolated microcapsules (T1 and T2) resulted in modest increases in antioxidant activity, which may be related to diffusional limitations imposed by the encapsulating matrix [28]. Lipid reformulation with oleogel (T3) was associated with increased antioxidant activity, which may be attributed to structural characteristics of the lipid network that can contribute to the protection of bioactive compounds [29,30,31].

The best performance was achieved with the hybrid system (T4), which had 55.7 µmol TE g^−1^, approximating the lower range of 70% cocoa chocolates. This result may indicate a synergistic effect, although the mechanisms involved require further investigation [32,33]. The strong correlation obtained between DPPH and β-carotene (r ≈ 0.9) suggests that antioxidant activity affects both electron- and hydrogen-transfer mechanisms [27]. However, the β-carotene assay appeared more sensitive to matrix modifications, which may reflect the contribution of lipophilic antioxidants such as tocopherols [26,34].

These results highlight that the concomitant engineering of the lipid phase and microencapsulation may represent a promising strategy to enhance antioxidant functionality without increasing cocoa content, aligning with the demand for functional chocolates with clean labeling.

## 5. Conclusions

This study evaluated the influence of extraction conditions and formulation strategies on the phenolic profile and antioxidant activity of dark chocolate. Among the tested solvents, 70% methanol showed the highest extraction efficiency, enabling broader recovery of phenolic compounds and alkaloids. Formulation T4, combining microencapsulation and lipid phase modification, exhibited the highest abundance of extractable compounds and the best antioxidant performance among the tested samples. These findings suggest that matrix modification strategies may enhance the extractability and functional expression of bioactive compounds in chocolate systems. However, the results should be interpreted with caution, as the mechanisms involved were not directly evaluated. Additionally, sensory properties were not assessed and represent an important limitation that should be addressed in future studies. Overall, this study contributes to the development of functional chocolate formulations with potential added value, highlighting the importance of matrix design in modulating bioactive compound behavior.

## Figures and Tables

**Table 1:** Percentage composition of ingredients in chocolate formulations (dry basis).

Ingredients %	C	T1	T2	T3	T4
**Cocoa liquor**	50.0	50.0	50.0	53.0	53.0
**Cocoa butter**	10.0	10.0	10.0	7.0	7.0
**Refined sugar**	39.2	39.1	29.0	26.2	26.1
**Soy lecithin**	0.5	0.5	0.5	0.5	0.5
**PGPR**	0.1	0.1	0.1	0.1	0.1
**Vanillin**	0.2	0.2	0.2	0.2	0.2
**Microcapsules (E/D/Zn)**	–	10.0	–	–	5.0
**Microcapsules (DHA/EPA + peanut oil)**	–	–	10.0	–	5.0
**Oleogel**	–	–	–	3.0	3.0

**Table 2:** Phenolic compounds and alkaloids identified by HPLC-DAD in chocolate samples extracted with 70% methanol.

Compounds	C	T1	T2	T3	T4
Vanillin	✓	✓	✓		✓
Vanillic acid	✓	✓	✓	✓	
Gallic acid	✓	✓	✓	✓	✓
Syringic acid	✓	✓	✓	✓	✓
*p*-Coumaric acid	✓	✓	✓	✓	✓
Caffeic acid	✓	✓	✓	✓	✓
Ferulic acid	✓	✓	✓	✓	✓
Luteolin	✓		✓	✓	✓
Quercetin	✓	✓	✓	✓	✓
Kaempferol	✓	✓		✓	✓
Epicatechin	✓	✓	✓	✓	✓
Epigallocatechin	✓	✓	✓	✓	✓
Caffeine	✓	✓	✓	✓	✓
Theophylline	✓	✓			
Theobromine	✓	✓	✓	✓	✓

**Table 3:** The phenolic profile obtained using 50% ethanol is presented.

Compounds	C	T1	T2	T3	T4
Vanillin	✓				
Vanillic acid	✓	✓			
Gallic acid	✓	✓	✓	✓	
Syringic acid	✓	✓		✓	✓
*p*-Coumaric acid	✓				✓
Caffeic acid	✓	✓		✓	✓
Ferulic acid	✓	✓	✓		
Luteolin	✓	✓			
Quercetin	✓		✓		
Kaempferol	✓				
Epicatechin	✓	✓		✓	
Epigallocatechin				✓	
Caffeine					
Theophylline	✓				
Theobromine	✓	✓	✓	✓	✓

**Table 4:** Phenolic compounds and alkaloids identified by HPLC-DAD in chocolate samples extracted with 70% acetone.

Compounds	C	T1	T2	T3	T4
Vanillin	✓	✓			
Vanillic acid					
Gallic acid	✓				
Syringic acid					
*p*-Coumaric acid					
Caffeic acid					✓
Ferulic acid	✓				
Luteolin					
Quercetin					
Kaempferol					
Epicatechin	✓				
Epigallocatechin					
Caffeine					
Theophylline					
Theobromine	✓	✓	✓	✓	✓

**Table 5:** Distribution of phenolic compound classes and alkaloids across formulations and extraction solvents (70% Methanol, 50% Ethanol, 70% Acetone), determined by HPLC-DAD. Data reflects combined effects of compound polarity, matrix accessibility, and solvent–matrix interactions. RT, retention time (minutes).

Class (RT)	Main Evidence	Discussion	References
**Hydroxybenzoic acids (4–7 min)**	Peaks detected mainly in methanolic extracts; fewer signals observed in ethanol and acetone.	These compounds present relatively high polarity due to hydroxyl and carboxyl groups, which favors extraction with hydroalcoholic solvents.	[18,19,20]
**Hydroxycinnamic acids (7–9 min)**	Signals observed mainly in methanolic extracts, with reduced recovery in ethanol and acetone systems.	The conjugated aromatic structure influences retention behavior and extraction efficiency depending on solvent polarity.	[19,20]
**Flavan-3-ols/procyanidins (9–14 min)**	Peaks predominantly observed in methanolic extracts.	Multiple hydroxyl groups increase polarity, favoring extraction in more polar solvent systems.	[20,21]
**Benzaldehyde derivatives (e.g., vanillin) (≈10–12 min)**	Peaks corresponding to aromatic aldehyde derivatives detected in the chromatographic profile.	These compounds are common aroma-related phenolics in cocoa matrices and may appear in intermediate retention regions.	[22]
**Flavonols/flavones (14–18 min)**	Signals mainly detected in methanol extracts and less frequently in ethanol extracts.	Polyhydroxylated aromatic structures generally require polar solvents for efficient extraction.	[15,20]
**Alkaloids (3–5 min)**	Early peaks consistent with methylxanthine-type compounds detected in the samples.	Cocoa naturally contains methylxanthines, which are stable compounds commonly observed in chromatographic analyses of cocoa products.	[23]

**Table 6:** Antioxidant Activity.

Treatment	DPPH (µmol TE g^−1^)	*β*-Carotene Bleaching (%)
**Control**	35.9 ± 1.0 ᵃ	40.0 ± 0.9 ᵃ
**T1**	38.5 ± 1.2 ᵃ	42.1 ± 1.4 ᵃ
**T2**	39.4 ± 0.9 ᵃ	43.3 ± 1.1 ᵃ
**T3**	47.1 ± 1.1 ᵇ	54.2 ± 1.2 ᵇ
**T4**	55.7 ± 1.3 ᶜ	64.9 ± 1.2 ᶜ

Identical letters in the column indicate no significant difference (Tukey, *p* > 0.05).

## Data Availability

The original contributions presented in the study are included in the article, further inquiries can be directed to the corresponding authors.
